# International Caries Detection and Assessment System: A new paradigm in detection of dental caries

**DOI:** 10.4103/0972-0707.53335

**Published:** 2009

**Authors:** KM Shivakumar, Sumanth Prasad, GN Chandu

**Affiliations:** Department of Public Health Dentistry, Sharad Pawar Dental College and Hospital, Datta Meghe Institute of Medical Sciences University, Sawangi (Meghe), Wardha - 42 004, Maharashtra, India; 1 Department of Public Health Dentistry, College of Dental Sciences, Davangere - 577004, Karnataka, India

**Keywords:** Dental caries, ICDAS, research

## Abstract

A new emphasis on caries measurement and management is required for the dental community. The dental professionals need new approaches in caries detection, its assessment, and management. The future of research, practice, and education in Cariology requires the development of an integrated definition of dental caries, and uniform systems for measuring the caries process. Keeping this in view, the International Caries Detection and Assessment System (ICDAS) has presented a new paradigm for the measurement of dental caries, which was developed from the systematic reviews of literature on the clinical caries detection system and other sources. The ICDAS can serve as a basis and benchmark for clinical and epidemiological research and inform dental undergraduate and postgraduate teaching in Cariology. The ICDAS system was developed to bring forward the current understanding of the process of initiation and progression of dental caries to the fields of epidemiological and clinical research.

## INTRODUCTION

The International Caries Detection and Assessment System (ICDAS) presents a new technique for the measurement of dental caries developed from the systematic reviews of literature on the clinical caries detection system and other sources.[1,2] All these reviews found that the new caries detection criteria measured different stages of the caries process. The review also found that there was a difference between different criteria for dental caries detection and there were many inconsistencies among the research criteria for measuring the caries.

All these systematic reviews suggested that there was an urgent need to answer the following questions:

1. What stage of the caries process should be measured?
2. What are the definitions for each selected stage?
3. What is the best clinical approach to detect each stage on different tooth surfaces?
4. What protocols of examiners' training can provide the highest degree of examiner reliability?

The ICDAS can serve as a basis and benchmark for clinical and epidemiological research and inform dental undergraduate and postgraduate teaching in Cariology. The members of the coordinating committee of ICDAS have attempted several times to include the largest input of the cariology community in the process of developing integrated criteria. There were various concepts debated on the field of Cariology, in the 1980s. The new emphasis on caries measurement and management may indicate that the dental community worldwide has started to recognize that we need new approaches in caries detection, assessment, and management.[3] The development of new technologies and applications has the potential to supplement clinical caries detection, but these assessments will have to be clinically meaningful by providing measurements over and above the rattle of the arrested initial and subclinical lesions .[4] 

## ICDAS: The committee

The ICDAS activities have been carried out under the supervision of and on behalf of an unfunded, informal, and an adhoc and voluntary committee, which was assembled in an attempt to advance some of the key recommendations in the area of caries detection and assessment criteria. After the first meeting in Dundee, Scotland, an invitation was mailed to cariologists from Europe and USA, to attend a development workshop in Ann Arbor, Michigan.[3]

## ICDAS: The philosophy

The philosophy on which this truly collaborative initiative is based on one where the methodology from the caries epidemiology meets the one from the caries clinical trials and practice, and the whole is conducted according to the values of Evidence Based Dentistry (EBD). The principles of the ICDAS committee are: integration, scientific validation, and utility of the criteria in different research and practice settings.[3]

## ICDAS: Developmental meetings

**Figure 1:**
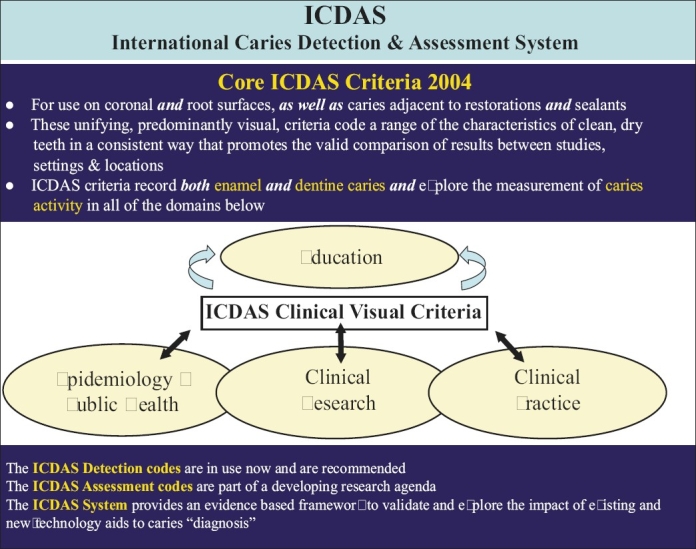
An overview of the development of the ICDAS

Before the ICDAS II workshop, four development meetings were held - Dundee, Scotland in April 2002; Ann Arbor, Michigan in August 2002; Indianapolis, Indiana in May 2003; and Bornholm, Denmark in April 2004. An overview of the development of the ICDAS, Denmark meeting (2004), is shown in Figure 1.

The ICDAS II workshop was held in Baltimore, USA in 2005, to share the progress in the ICDAS criteria and seek the input of a wider international expertise. Invitations were mailed to a large group of experts and those who accepted the invitation convened to review, revise as necessary, and agree upon the ICDAS II version of the criteria. The invitations were mailed to over 60 cariologists and researchers in the field.[3]

## ICDAS: The concepts

A “wardrobe” of validated tools should allow users to select the best criteria and conventions for a specific use. The concept is that the system will be an open one maintained on the World Wide Web and subject to peer review. Users of the system will have to: specifically acknowledge the version of the system they employ and specify the parts of the “ICDAS wardrobe” that are being used. 

## CORON AL PRIMARY CARIES DETECTION CRITERIA

### Principles used to develop the criteria for coronal primary caries

Dental caries is a dynamic process with cycles of demineralization followed by remineralization. It is hard to categorize a complex disease like dental caries into a scale because the process is continuous and can be measured. Clinically, we rely on visual signs that represent manifestations of a relatively advanced caries process.[3]

The ICDAS measures the surface changes and potential histological depth of the carious lesions by relying on surface characteristics. The primary requirement for applying the ICDAS system is the examination of clean and dry teeth. The ICDAS examination is visually aided by a ball-ended explorer that is used to remove any remaining plaque and debris and to check for surface contour, minor cavitation or sealants. It is highly advisable that the teeth are cleaned with a toothbrush or a prophylaxis head/cup before the clinical examination. The use of a sharp explorer is not necessary because it does not add to the accuracy of the detection and it may damage the enamel surface covering the early carious lesions.[5]

The ICDAS detection codes for coronal caries range from 0 to 6 depending on the severity of the lesion. There are minor variations between the visual signs associated with each code depending on a number of factors, including the surface characteristics; whether there are adjacent teeth present and also whether or not the caries are associated with a restoration or sealant. Therefore, a detailed description of each of the codes is given under the following headings, to assist in the training of examiners in the use of ICDAS: Pits and fissures; smooth surface (mesial or distal); free smooth surfaces and caries associated with restorations and sealants (CARS).[6] However, the basis of the codes is essentially the same throughout:

**Table:** 

Code	Description
0	Sound
1	First visual change in enamel (seen only after prolonged air drying or restricted to the confines of a pit or fissure)
2	Distinct visual change in enamel
3	Localized enamel breakdown (without clinical visual signs of dentinal involvement)
4	Underlying dark shadow from dentin
5	Distinct cavity with visible dentin
6	Extensive distinct cavity with visible dentin

#### A. Pits and fissure caries

*Code 0: Sound tooth surface:* There should be no evidence of caries. Surfaces with developmental defects such as enamel hyperplasia, fluorosis, tooth wear (attrition, abrasion, and erosion), and extrinsic or intrinsic stains will be recorded as sound. The examiner should also score as sound, a surface with multiple stained fissures if such a condition is seen in other pits and fissures.

* Code 1: First visual change in enamel:* When seen wet there is no evidence of any change in color attributable to carious activity, but after prolonged air drying, a carious opacity or discoloration (white or brown lesion) is visible, which is not consistent with the clinical appearance of sound enamel, or when there is a change of color due to caries it is not consistent with the clinical appearance of sound enamel and is limited to the confines of the pit and fissure area (whether seen wet or dry). The appearance of these carious areas is not consistent with that of stained pits and fissures as defined in code 0. 

* Code 2: Distinct visual change in enamel:* The tooth must be viewed wet. When wet there is a carious opacity (white spot lesion) and/or brown carious discoloration that is wider than the natural fissure/fossa, which is not consistent with the clinical appearance of sound enamel.

* Code 3: Localized enamel breakdown due to caries with no visible dentin or underlying shadow:* The tooth viewed wet may have a clear carious opacity (white spot lesion) and/or brown carious discoloration that is wider than the natural fissure/fossa, which is not consistent with the clinical appearance of sound enamel. Once dried, there is carious loss of tooth structure at the entrance to, or within the pit or fissure/fossa. This will be seen visually as evidence of demineralization at the entrance to or within the fissure or pit, and although the pit or fissure may appear substantially and unnaturally wider than normal, the dentin is not visible in the walls or base of the cavity/discontinuity. 

If in doubt, or to confirm the visual assessment, the WHO/CPI/PSR probe can be used gently across the tooth surface, to confirm the presence of a cavity apparently confined to the enamel. This is achieved by sliding the ball end along the suspect pit or fissure and a limited discontinuity is detected if the ball drops into the surface of the enamel cavity/discontinuity.

* Code 4: An underlying dark shadow from dentin with or without localized enamel breakdown:* This lesion appears as a shadow of discolored dentin visible through an apparently intact enamel surface, which may or may not show signs of localized breakdown. The shadow appearance is often seen more easily when the tooth is wet. The darkened area is an intrinsic shadow that may appear gray, blue or brown. The shadow must clearly represent caries that started on the tooth surface being evaluated. If in the opinion of the examiner, the carious lesion started on an adjacent surface and there was no evidence of any caries on the surface being scored, then the surface should be coded “0”. Codes 3 and 4, histologically may vary in depth with one being deeper than the other and vice versa. 

* Code 5: Distinct cavity with visible dentin:* Cavitation in opaque or discolored enamel, exposing the dentin beneath. The tooth viewed wet may have darkening of the dentin visible through the enamel. Once dried, there is visual evidence of loss of tooth structure at the entrance to or within the pit or fissure - frank cavitation. There is visual evidence of demineralization (opaque (white), brown or dark brown walls) at the entrance to or within the pit or fissure and in the examiner's judgment, the dentin is exposed.

The WHO/CPI/PSR probe can be used to confirm the presence of a cavity, apparently in the dentin. This is achieved by sliding the ball end along the suspect pit or fissure and a dentin cavity is detected if the ball enters the opening of the cavity and in the opinion of the examiner the base is in dentin. (In pits or fissures the thickness of the enamel is between 0.5 and 1.0 mm. Note the deep pulpal dentin should not be probed) 

* Code 6: Extensive distinct cavity with visible dentin:* There is obvious loss of tooth structure, the cavity is both deep and wide, and the dentin is clearly visible on the walls and at the base. An extensive cavity involves at least half of a tooth surface or possibly reaches the pulp.

#### B. Smooth surface caries[6]

This requires visual inspection from the occlusal, buccal, and lingual directions.

* Code 0: Sound tooth surface:* There should be no evidence of caries. Surfaces with developmental defects such as enamel hyperplasia, fluorosis, tooth wear (attrition, abrasion and erosion), and extrinsic or intrinsic stains will be recorded as sound. 

* Code 1: First visual change in enamel:* When seen wet there is no evidence of any change in color attributable to carious activity, but after prolonged air drying a carious opacity (white or brown lesion) is visible that is not consistent with the clinical appearance of sound enamel. This will be seen from the buccal or lingual surface.

* Code 2: Distinct visual change in enamel when viewed wet:* There is a carious opacity or discoloration (white or brown lesion) that is not consistent with the clinical appearance of sound enamel. This lesion may be seen directly when viewed from the buccal or lingual direction. In addition, when viewed from the occlusal direction, this opacity or discoloration may be seen as a shadow confined to enamel, seen through the marginal ridge.

* Code 3: Initial breakdown in enamel due to caries with no visible dentin:* Once dried for approximately five seconds there is distinct loss of enamel integrity, viewed from the buccal or lingual direction. If in doubt, or to confirm the visual assessment, the CPI probe can be used gently across the surface to confirm the loss of surface integrity.

* Code 4: Underlying dark shadow from dentin with or without localized enamel breakdown:* This lesion appears as a shadow of discolored dentin visible through an apparently intact marginal ridge, buccal or lingual walls of enamel. This appearance is often seen more easily when the tooth is wet. The darkened area is an intrinsic shadow which may appear as gray, blue or brown.

* Code 5: Distinct cavity with visible dentin:* Cavitation in opaque or discolored enamel (white or brown) with exposed dentin in the examiner's judgment. If in doubt, or to confirm the visual assessment a CPI probe is used to confirm the presence of a cavity apparent in dentin. This is achieved by sliding the ball end along the surface and a dentin cavity is detected if the ball enters the opening of the cavity and in the opinion of the examiner the base is in dentin.

* Code 6: Extensive distinct cavity with visible dentin:* Obvious loss of tooth structure, the extensive cavity may be deep or wide and dentin is clearly visible on both the walls and at the base. The marginal ridge may or may not be present. An extensive cavity involves at least half of a tooth surface or possibly reaching the pulp. 

### CARIES ADJACENT TO RESTOR ATIONS AND SEALANTS (CARS)

When a restoration is placed in a tooth, the adjacent tooth tissue, which is vulnerable to caries, can be considered in two planes. Secondary caries has classically been described as occurring in two ways: an “outer lesion” and a “wall lesion”. The chemical and histological processes involved in "outer lesions" are the same as primary caries.[7]

### Principles used to develop the criteria for CARS

Since “outer” carious lesions adjacent to restorations are thought to be analogous with primary caries, the broad principles applied to the criteria for primary caries are also applied to CARS where relevant. However, it should be noted that the scientific basis for doing so has not been established and the literature in the area of secondary caries is far more limited than that for primary coronal caries.[3]

### Caries associated with restoration and sealant codes[6]

*Code 0:* Sound tooth surface with restoration or sealant

* Code 1:* First visual change in enamel

* Code 2:* Distinct visual change in enamel/dentin adjacent to a restoration/sealant margin

* Code 3:* Carious defects of < 0.5 mm, with signs of code 2 

* Code 4:* Marginal caries in enamel/dentin/cementum adjacent to restoration/sealant, with underlying dark shadow from dentin

* Code 5:* Distinct cavity adjacent to restoration/sealant

* Code 6:* Extensive distinct cavity with visible dentin

### ICDAS two-digit coding method

A two-number coding system is suggested to identify restorations/sealants with the first digit, followed by the appropriate caries code, for example, a tooth restored with amalgam, which also exhibits an extensive distinct cavity with visible dentin will be coded 4 (for an amalgam restoration) and 6 (for a distinct cavity); an unrestored tooth with a distinct cavity would be 06. The suggested restoration/sealant coding system is as follows:[6]

0 = Sound, that is, surface not restored or sealed (use with the codes for primary caries)

1 = Sealant, partial

2 = Sealant, full

3 = Tooth colored restoration

4 = Amalgam restoration

5 = Stainless steel crown

6 = Porcelain or gold or PFM crown or veneer

7 = Lost or broken restoration

8 = Temporary restoration

9 = Used for the following conditions

96 = Tooth surface cannot be examined: surface excluded

97 = Tooth missing because of caries (tooth surfaces will be coded 97)

98 = Tooth missing for reasons other than caries (all tooth surfaces will be coded 98)

99 = Unerupted (tooth surfaces coded 99)

## ROOT CARIES

According to the National Institutes of Health (NIH) Consensus Development Conference on dental caries diagnosis and management, it was concluded that there was “insufficient” evidence on the validity of clinical diagnostic systems for root caries.[8] Root caries are frequently observed near the cemento-enamel junction (CEJ), although lesions can appear anywhere on the root surface. The color of the root lesions has been used as an indication of lesion activity. Active lesions have been described as being yellowish or light brown in color, whereas, arrested lesions appear darkly stained. However, color subsequently has been shown not to be a reliable indicator of caries activity.[9,10]

## Codes for the detection and classification of carious lesions on the root surfaces[6]

One score will be assigned per root surface. The facial, mesial, distal, and lingual root surfaces of each tooth should be classified as follows:

* Code E:* If the root surface cannot be visualized directly as a result of gingival recession or by gentle air-drying, then it is excluded. Surfaces covered entirely by calculus can be excluded or, preferably, the calculus can be removed prior to determining the status of the surface. 

* Code 0:* The root surface does not exhibit any unusual discoloration that distinguishes it from the surrounding or adjacent root areas, nor does it exhibit a surface defect either at the CEJ or wholly on the root surface. The root surface may have a natural anatomical contour or the root surface may exhibit a definite loss of surface continuity or an anatomical contour that is not consistent with the dental caries process.

**Figure 2:**
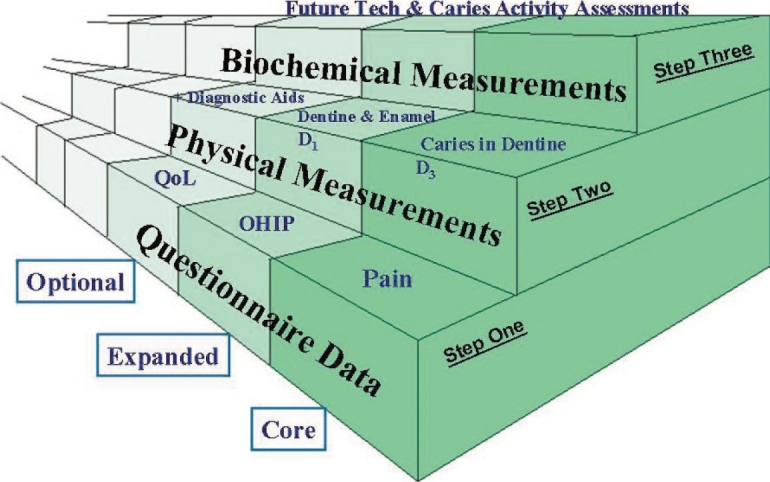
Adaptation of the WHO's “stepwise” approach to the surveillance of noncommunicable diseases for use with oral health indicators. [Courtesy from: Pitts NB ICDAS - an international system for caries detection and assessment being developed to facilitate caries epidemiology, research and appropriate clinical management. Community Dental Health 2004;21:193–198.]

* Code 1:* There is a clearly demarcated area on the root surface or at the CEJ that is discolored (light/dark brown, black) but there is no cavitation (loss of anatomical contour < 0.5 mm) present.

* Code 2:* There is a clearly demarcated area on the root surface or at the CEJ that is discolored (light/dark brown, black) and there is cavitation (loss of anatomical contour ≥ 0.5 mm) present.

## THE FUTURE OF ICDAS

Researchers and clinicians have chosen the stage of disease and characteristics for assessment of carious lesions. [Figure 2] shows the adaptation of WHO “stepwise” approach to the surveillance of noncommunicable diseases for use with oral health indicators. The STEPS approach allows a logical organization of different and often disparate indicators used, into a series of core indicators that can be used at STEP 1, 2 or 3, depending on the circumstances and local needs, preferences. and resources. This philosophy is entirely consistent with the wardrobe approach of ICDAS and its use would result in the improved comparability of data collected nationally and internationally, thereby, facilitating systematic reviews in the area.[3,11] The future of ICDAS depends on the acceptance of the concepts of integration and utility within a caries detection and assessment system.[11]
